# Ozone efficacy for the control of airborne viruses: Bacteriophage and norovirus models

**DOI:** 10.1371/journal.pone.0231164

**Published:** 2020-04-10

**Authors:** Marie-Eve Dubuis, Nathan Dumont-Leblond, Camille Laliberté, Marc Veillette, Nathalie Turgeon, Julie Jean, Caroline Duchaine

**Affiliations:** 1 Centre de Recherche de l’Institut Universitaire de Cardiologie et de Pneumologie de Québec – Université Laval, Quebec City, Quebec, Canada; 2 Département de Biochimie, de Microbiologie et de Bio-informatique, Faculté des Sciences et de Génie, Université Laval, Quebec City, Quebec, Canada; 3 Département des Sciences des Aliments, Faculté des Sciences de l’Agriculture et de l’Alimentation, Université Laval, Quebec City, Quebec, Canada; Valahia University of Targoviste, ROMANIA

## Abstract

This study was designed to test the efficacy of an air treatment using ozone and relative humidity (RH) for the inactivation of airborne viruses. Four phages (φX174, PR772, MS2 and φ6) and one eukaryotic virus (murine norovirus MNV-1) were exposed to low ozone concentrations (1.23 ppm for phages and 0.23 ppm for MNV-1) and various levels of RH for 10 to 70 minutes. The inactivation of these viruses was then assessed to determine which of the tested conditions provided the greatest reduction in virus infectivity. An inactivation of at least two orders of magnitude for φX174, MS2 and MNV-1 was achieved with an ozone exposure of 40 minutes at 85% RH. For PR772 and φ6, exposure to the reference condition at 20% RH for 10 minutes yielded the same results. These findings suggest that ozone used at a low concentration is a powerful disinfectant for airborne viruses when combined with a high RH. Air treatment could therefore be implemented inside hospital rooms ventilated naturally.

## Introduction

Viral infections can be acquired in numerous indoor public spaces, including hospitals, cruise ships, schools, daycare centres, restaurants, and transport and commuting services [1, 2]. Evidence for the presence of multiple viruses in these settings, including influenza, rhinovirus, coronavirus, adenovirus, enterovirus, norovirus and the respiratory syncytial virus (RSV) have been reviewed [3]. Infections acquired in hospital settings are a major concern for patients, workers and visitors. They are responsible for longer hospital stays [4], increased costs [4–8], absenteeism among healthcare workers [4], and even patient deaths [9]. Norovirus, influenza, rotavirus and RSV are among the most common viruses acquired in hospital settings [10, 11]. On various occasions, cruise ships have been struck by norovirus outbreaks, infecting hundreds of people at once [12–18]. As mentioned by Lopman *et al*. (2012) [1], norovirus has also been problematic in other indoor environments, including restaurants, schools and kindergartens, concert halls, airplanes and buses.

Viruses are transmitted through multiple routes [3], including transmission through contact, transmission by a vehicle (water, food, fomites or inanimate objects) or a vector (insects) and finally airborne transmission [19]. Large aerosol droplets usually travel shorter distances, generally a few dozen centimetres [19, 20]. Aerosols of smaller size can habitually remain in the air for longer time periods and consequently can travel over long distances (more than 1 m) [3, 19–21]. Bioaerosols can also settle after a prolonged time period, leading to fomite contamination [22]. A second aerosolization from these contaminated fomites is also possible and may cause further propagation of pathogens [3, 23].

The airborne transmission route has been proven to facilitate the transmission of tuberculosis [24], respiratory viruses such as influenza and rhinoviruses, gastrointestinal viruses such as rotavirus [20], and is suspected of playing a role in the transmission of other pathogens such as norovirus [19]. According to Jones and Brosseau (2015) [19], the biological plausibility of aerosol transmission for norovirus is scored at seven out of nine, which indicates that aerosol transmission for this pathogen is of great concern. Moreover, some authors suggest that gastrointestinal viruses may enter the body through the respiratory tract [19, 25–27] and can then be swallowed, leading to infection.

Depending on the pathogen’s route, transmission of viral diseases in indoor settings can be controlled through various procedures including the use of personal protective equipment [28]. Surface disinfection protocols are already in place in hospitals, airplanes, schools and daycare facilities. It is well documented that a sodium hypochlorite solution (bleach) is an effective way to inactivate norovirus [29–31], while alcohol-, detergent- and quaternary ammonium compound-based disinfectants have more limited effects [27, 29, 32–34]. Since sodium hypochlorite is corrosive and an irritant to mucous membranes, skin and airways [35], it is usually employed only for specific tasks or during outbreaks [36]. As for disinfection time, Tuladhar et al. (2012) [37] suggest that sodium hypochlorite should be in contact with surfaces for 5 min for reducing norovirus, while the World Health Organization recommends a contact time of at least 10 min for this disinfectant, regardless of the pathogen [35]. Even if the recommendations differ, these contact times are often difficult to achieve because of the workload and availability of environmental hygiene personnel. The personal protective equipment (PPE) that is recommended during viral outbreaks includes disposable gowns, gloves, respirators and even eye protection [31] when working in hospitals, though they are rarely worn in other indoor public spaces. Unfortunately, the efficacy of interventions for reducing the transmission and inhibiting the development of infections has not yet been established [10, 30, 33, 38].

Because norovirus is a highly resistant, persistent and stable virus [1, 34, 39–41] that is still infectious when airborne [42], air treatment should be considered to reduce infectivity and further contamination of fomites and other objects.

Currently, there are no air treatment strategies available for inactivating airborne viruses during viral hospital outbreaks, which is due to the lack of approved protocols. UV light, ozone and disinfecting agents have been tested for airborne phage and virus inactivation [43–46], but none of them have led to the establishment of standardized air treatment protocols. In addition, they were used for short periods of time (≤ 1 minute) and many were at high concentrations that are toxic for humans. Such treatments could be used in the heating and ventilation plenums to inactivate viruses. However, since some hospital rooms are not mechanically ventilated, another strategy could be the implementation of an air treatment during times of no occupancy, when hospital rooms are vacant.

For this study, we selected ozone as the disinfecting agent because it is in a gas state at room temperature and it has proven virucidal properties [47]. As mentioned by Hudson *et al*. (2007) [48], the gas state allows ozone to get to areas that are difficult to reach and to disinfect much more than just surfaces. The Immediately Dangerous to Life or Health Concentration (IDLH) of ozone is 5 ppm for humans. In order to protect the health of occupants, and keeping in mind that leakage from the closed hospital rooms can occur, it is crucial that the concentration used for air treatment be below this value. In the literature, ozone concentrations between 6.25 ppm and 60,000 ppm have been used [47–51] for inactivating norovirus surrogates on surfaces or food, which are all above the IDLH. Three studies used concentrations below the IDHL, with exposure times of 2 min or less [52–54]. Our study was designed to use lower ozone concentrations but for longer exposure periods in order to treat the air in unoccupied and unsealed rooms [55, 56].

Model phages have been developed and used as surrogates for eukaryotic viruses [45, 57, 58] because they are easier to work with and are non-pathogenic to humans, requiring less extensive containment facilities. It is important to use multiple phages with different features (e.g. with and without an envelope, RNA and DNA, single and double-stranded) to represent a broader range of eukaryotic viruses and their resistance when airborne and when exposed to disinfecting agents. For this reason, four model phages were selected: MS2, φ6, PR772 and φX174. MS2 is a widely recognized model for norovirus [59, 60]. Because of its envelope, φ6 is considered to be a good surrogate for Influenza. PR772 is a good model for the human adenovirus [61]. Lastly, φX174 was selected for its genetic material (single-stranded DNA phage) and ease of use. In addition to the phages, a eukaryotic virus, MNV-1, was selected. MNV-1 is a murine surrogate for human norovirus, the latter cannot be cultivated *in vitro* [34]. Its characteristics and behaviour are similar to the human norovirus and it can be replicated in cell culture, making MNV-1 the most widely accepted surrogate [34, 62, 63].

The aim of this study was to investigate the effect of low ozone concentration on four phage models and on MNV-1 using low (20%), medium (55%) and high (85%) relative humidity in order to evaluate the feasibility of ozone disinfection in hospital rooms using passive ventilation. When ozone reacts with water, it forms free radicals that can increase disinfection power: the superoxide anion (·O_2_-), the hydroxyperoxyl radical (HO_2_·) and the hydroxyl radical (·OH) [46]. Our hypothesis, supported by Hudson *et al*. (2007) [48] and Li and Wang’s (2003) [64] work, is that more free radicals will be formed when air humidity is higher, which could lead to higher virus inactivation. Three exposure times (10, 40 and 70 minutes) were also selected to verify whether virus infectivity decreases over time.

## Materials and methods

### Model phages and host bacteria

Four model phages and their respective host bacteria were used for this study: φ6, φX174, PR772, and MS2. All phages and host strains were provided by the Félix d’Hérelle Reference Center for Bacterial Viruses. Their characteristics and growth conditions are listed in Table 1. Phage lysate from a second amplification of each phage was used to constitute the viral stock that was then nebulized. Phages φ6 and PR772 were amplified on Tryptic Soy Agar (TSA) media using the soft agar method (0.75% agar). Tryptic Soy Broth (TSB) was used for amplification of phages φX174 and MS2.

**Table 1:** Bacteria and phages.

Bacterial or viral strains	Characteristics	Growth conditions	Bacterial host	References
HER1036	*Escherichia coli*	TSB, 37°C, 200 rpm	-	[65]
HER1102	*Pseudomonas syringae* var. *phaseolicola*	TSB, 25°C, 100 rpm	-	[66]
HER1221	*E*. *coli*	TSB, 37°C, 200 rpm	-	[67]
HER1462	*E*. *coli*	TSB, 37°C, 200 rpm	-	[66]
HER36	Phage φX174, 25 nm, nonenveloped, linear ssDNA, 5386 bases	-	HER1036	[65]
HER102	Phage φ6, 85 nm, enveloped, segmented dsRNA, 13385 bp	-	HER1102	[66]
HER221	Phage PR772, 80 nm, nonenveloped, linear dsDNA, 14492 bp	-	HER1221	[67]
HER462	Phage MS2, 25 nm, nonenveloped, linear ssRNA, 3569 bases	-	HER1462	[66]

ssDNA: single stranded DNA, ssRNA: single stranded RNA, dsDNA: double-stranded DNA, dsRNA: double-stranded RNA, bp: base pairs

### MNV-1 and host cells

MNV-1 (PTA-5935) and host cells (RAW 264.7; TIB-71) were purchased from ATCC. MNV-1 was amplified using host cells following the Wobus *et al*. (2004) [62] protocol. Viral stock containing approximately 1 X 10^7^ PFU/ml (viruses in Dulbecco modified Eagle’s medium (DMEM) + 10% FBS) was divided into 30 ml volumes and placed in conical plastic tubes and stored at -80°C until nebulization.

### Environmental aerosol chamber setup

**Fig 1:**
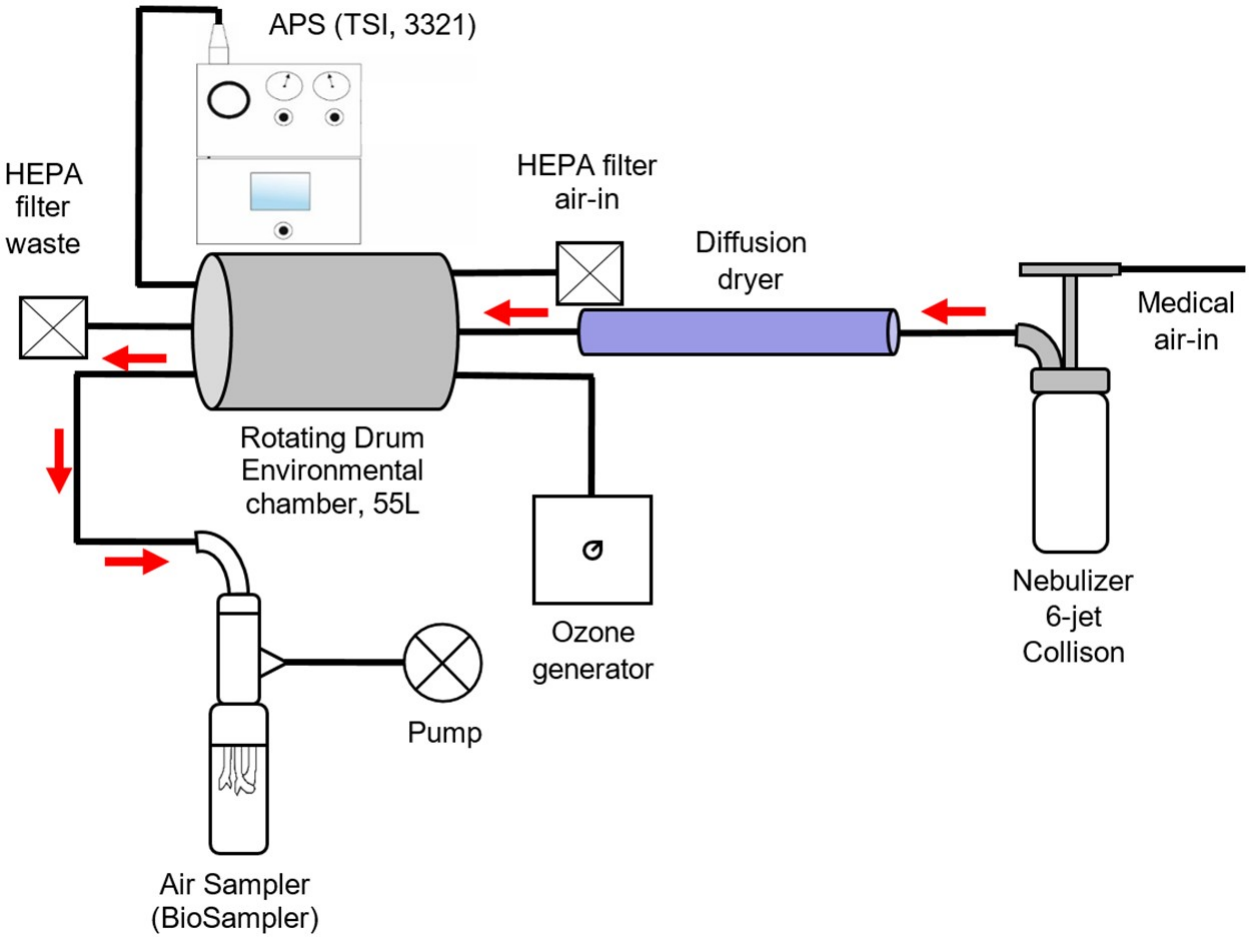
Complete environmental aerosol chamber set up.

Viruses were aerosolized into a rotative environmental aerosol chamber that is also called a Golberg drum or rotating drum [68]. The chamber was enclosed in a biosafety level II cabinet to ensure the containment of viruses in case of leakage. The drum rotation speed was set at 1 rpm to ensure that aerosols remained in suspension. Fig 1 depicts the complete set up.

An ozone generator (model EMO3-VTTL, EMO_3_, Quebec City, CANADA) was connected to the chamber. The concentration was assessed using a 37.8 L calm-air chamber and an ozone probe that collects real-time readings (model K60-O_3_ #600335; Nanjing Kelisaike Safety Equipment CO. LTD., Nanjing, CHINA). For phage exposure, ozone and air (control) were injected for 25 seconds at a flow rate of 0.4 L/min that was previously adjusted using a flowmeter (model 4140, TSI Inc., Shoreview, USA). We used a smaller ozone generator (model 201705004A210Y, EMO_3_) to examine MNV-1 exposure. The gas was injected for 30 seconds at a flow rate of 0.4 L/min. Therefore, the ozone concentrations used for phage and MNV-1 exposure were 1.13 ± 0.26 ppm and 0.23 ppm ± 0.03 ppm, respectively. The latter represents the lowest reproducible concentration that could be obtained with the experimental set-up.

An ozone destructor was placed inside the biosafety cabinet to protect the operator in case of leakage. An Aerosol Particle Sizer (APS) (model 3321; TSI Inc., Shoreview, USA) coupled with a 1/100 dilutor mounted with a 1/20 capillary (model 3302A, TSI Inc.) was used to track the size and the numbers of particles generated. The temperature and relative humidity (RH) inside the chamber were monitored with a probe (model TH-210, KIMO Instruments, Montpon, FRANCE).

### Phage and MNV-1 aerosolization

Each experiment was conducted in triplicate. A volume of 35 ml of phage buffer 1X (20 mM tris, 100 mM NaCl and 10 mM MgSO_4_) containing between 4.4 X 10^6^ and 1 X 10^8^ plaque forming units per millilitre (PFU/ml) of each phage and 5 μl of Antifoam A concentrate (Sigma-Aldrich, St-Louis, USA) was placed in a 6-jet Collison (BGI, Waltham, USA) supplied with 20 psi of compressed air (medical grade) and nebulized for 10 minutes. For MNV-1, 30 ml of viral stock (between 3.3 X 10^5^ and 4.4 X 10^6^ PFU/ml) that had been frozen at -80°C were thawed and placed in the nebulizer. Aerosols were forced through diffusion dryers of different lengths (327.4 cm, 203.7 cm, 37.7 cm) before they entered the chamber, in order to control the RH and achieve 20%, 55% and 85%, respectively. The particles generated had a mass median aerodynamic diameter (MMAD) of 1.10 ± 0.03 μm at 20% RH, 1.27 ± 0.03 μm at 55% RH and 1.24 ± 0.04 μm at 85% RH. The targeted temperature inside the rotating drum was 19°C ± 1°C. Aerosols were mixed for 10 minutes in the drum to achieve an even distribution before particles were counted using an APS. Ozone or air (reference condition) was injected into the chamber, and the aerosols were in contact with the gas for 0, 30 or 60 minutes before the air was sampled.

### Aerosol sampling

Samples were collected after a 0-, 30- or 60-minute exposure to air or ozone. The BioSampler (SKC Inc., Eighty Four, USA) was chosen for its great recovery of particles measuring 1 μm. Since particle’s MMAD was between 1.1 and 1.3 μm, it was assumed that the collection efficiency would be similar for all experiments. This sampler was filled with 20 ml of phage buffer or DMEM (for MNV-1) and connected to a SKC vacuum pump (model 228 ± 9605; SKC Inc.) to obtain a flow rate of 12.5 L/min, as determined by the critical orifice of the instrument. The sampler was operated for 20 minutes, which is the length of time required to empty the chamber.

Since viruses were sampled over a 20-minute period, the additional exposure times of individual particles to air or ozone varied between 0 and 20 minutes. Therefore, 10 minutes (or half of this range), was added to the previously described exposure times, representing the mean exposure during sampling. Therefore, the new exposure times were calculated to be 10, 40 and 70 minutes.

Control samples were taken from the nebulizing stock before the first and between each nebulization in order to monitor the variation in virus concentrations after each nebulization. A total of n+1 (where n = number of nebulizations performed in a day) control samples were collected. Samples and control samples were then quantified by plaque assay and qPCR. For qPCR, samples were kept at -20°C (phages) or -80°C (MNV-1) until analysis.

### Quantification of phages using plaque assay

Samples and controls were diluted (using 10-fold serial dilutions) with phage buffer to achieve the desired concentration for each of the four phages. Plaque assays were performed on TSA Petri dishes using the soft agar method and the host bacterial cells. Briefly, 100 μl of the required phage dilution was mixed with 100 μl of host bacterial cells that were grown overnight, in 3 ml of TSB soft agar (0.75%). The inoculated soft agar was then poured over a TSA Petri dish. When solidified, Petri dishes were incubated for 24h at 25°C for φ6 and 37°C for φX174, PR772 and MS2. Plaques were quantified after the incubation period and concentration calculations were performed to obtain the amount of PFU/ml.

### Quantification of MNV-1 using plaque assay

Quantification of infectious viruses were performed on host cells using the plaque forming unit method in 6-well plates. Host cells were grown in T-75 flasks (Corning, Corning, USA) in DMEM (Wisent, Saint-Jean-Baptiste, CANADA) + 10% FBS (Wisent) at 37°C + 5% CO_2_ until a confluence of 90% was reached. Cells were then passed and seeded in 6-well plates (Corning, Kennebunk, USA). Plates were incubated for 24 h prior to the infection step.

Serial dilutions of the samples were performed in PBS 1X until a desired concentration range was achieved (10^0^ to 10^−4^ for ozone and air samples and 10^−3^ to 10^−7^ for control samples). From the control samples, only the first and the last were quantified. Volumes of 750 μl of the appropriate dilutions were poured into each well containing a monolayer of host cells. Each dilution was performed in triplicate. Plates were placed at 37°C + 5% CO_2_ for 90 minutes to ensure proper infection. After the infection period, 6 ml of a 50/50 mix of SeaPlaque agarose (Lonza, Rockland, USA) 1.6% and 2X DMEM +20% FBS were poured over the cells. Once jellified at room temperature, this mix formed a solid plug, trapping the multiplying viruses and forcing them to infect adjacent cells, leading to the formation of plaques. Plates were incubated for 60 h at 37°C + 5% CO_2_ followed by a fixation step using 3.7% formaldehyde (37% formaldehyde diluted in distilled water) and a colouring step using a 0.8% crystal violet solution (0.8 g crystal violet in 100 ml distilled water). For the phages, plaques were quantified and PFU/ml was determined.

### Extraction of phage and MNV-1 RNA

For MNV-1 and phages φ6 and MS2, RNA extraction was performed using the QIAamp Viral RNA mini kit (QIAGEN, Hilden, GERMANY). Samples were eluted in two volumes of 40 μl of TE buffer (10 mM Tris, 0.1 mM EDTA), for a total of 80 μl and no RNA carrier was used. RNA was then stored at -80°C until cDNA synthesis.

### Quantification of phages and MNV-1 genomes by qPCR

#### Phage and MNV-1 genome cDNA synthesis

RNA was heated at 100°C for 5 minutes prior to cDNA synthesis. A volume of 5 μl was converted to cDNA using an iScript cDNA Synthesis Kit (Bio-Rad, Hercules, USA) according to the manufacturer’s instructions.

#### qPCR quantification

Primers and probes (Integrated DNA Technologies, Coralville, USA) used for phage and MNV-1 quantification are presented in Table 2. Each reaction mixture (total volume of 20 μl) contained the following: 10 μl of IQ Supermix (Bio-rad), 2 μl of cDNA (MS2, φ6 and MNV-1) or 5 μl of the sample (φX174 and PR772), 1 μM of forward and reverse primers and 150 nM (MS2 and φ6) or 200 nM (φX174, PR772 and MNV-1) of probe. The following amplification protocol was used for each of the phages: 95°C for 5 minutes then 39 cycles at 95°C for 15 seconds, 60°C for 60 seconds followed by a fluorescence reading. The Girard *et al*. (2010) [69] protocol was used for quantification of MNV-1: 95°C for 5 minutes, followed by 40 cycles at 95°C for 15 seconds, 58°C for 60 seconds and then a fluorescence reading. A 10-fold dilution series standard curve of plasmid DNA was used for each phage and MNV-1. A volume of 2 μl of cDNA was used to quantify φ6 and MS2. For MNV-1, 2 μl of cDNA were used as well, although it was diluted to 1/100 for exposed virus and 1/10 000 for controls in order to fit the standard curve. Volumes of 5 μl of raw samples diluted from 1/10 to 1/1000 were used for DNA phages (φX174 and PR772). DNA amplification was performed using the Bio-Rad CFX384 thermocycler (Bio-Rad Laboratories, Mississauga, CANADA). No template controls (NTC) were used as negative controls for qPCR. All NTC cycle threshold (CT) values were higher than the last standard curve value for each virus (φX174: 10 copies, PR772: 10 copies, MS2: 100 copies, φ6: 10 copies and MNV-1: 1000 copies). Extraction blanks for RNA viruses (MS2, φ6 and MNV-1) were also subtracted from samples. Extraction was not required for DNA phages, therefore no other controls were performed.

**Table 2:** Primers and probes.

Virus	Forward primers	Reverse primers	Probes	References
φX174	ACA AAG TTT GGA TTG CTA CTG ACC	CGG CAG CAA TAA ACT CAA CAG G	FAM-CTC TCG TGC-ZEN-TCG TCG CTG CGT TGA-IABlkFQ	[65]
φ6	TGG CGG CGG TCA AGA GC	GGA TGA TTC TCC AGA AGC TGC TG	FAM-CGG TCG TCG-ZEN-CAG GTC TGA CAC TCG C-IABlkFQ	[66]
PR772	CCT GAA TCC GCC TAT TAT GTT GC	TTT TAA CGC ATC GCC AAT TTC AC	FAM-CGC ATA CCA-ZEN-GCC AGC ACC ATT ACG CA-IABlkFQ	[57]
MS2	GTC CAT ACC TTA GAT GCG TTA GC	CCG TTA GCG AAG TTG CTT GG	FAM-ACG TCG CCA-ZEN-GTT CCG CCA TTG TCG-IABlkFQ	[66]
MNV-1	GCT GCG GCC TCT CTT GAC	AGG GAT GGT GTC CTG AAA ACC	6FAM-TTC GTG CGG TCC CAA GAT CCA TCT-TAMRA	[69]

### Ozone effect in air sampler

Control experiments for ozone in air samplers were performed to verify if ozone has an effect within the collection liquid of the air sampler. Indeed, because ozone has virucidal properties in water, the quantification of this effect had to be assessed so that a mathematical correction could be applied. It has also been previously found that ozone had an effect on MS2 infectivity in a liquid impinger [70]. Experiments were performed in duplicate.

Biosampler collection fluid (phage buffer 1X) was spiked with 1 X 10^7^ PFU/ml of each phage. Phage buffer 1X was nebulized to humidify the rotating chamber. Duplicates of 55% and 29% RH were performed. Ozone was then injected into the drum followed by air sampling. Air was used instead of ozone as a control. The collection fluid was diluted (10 fold serial dilutions) with phage buffer 1X and then quantified by plaque assay and qPCR.

### Calculations

$${\text{IR}}_{\text{air}}=<\text{PFU}/\text{ml}>/<\text{genomes}/\text{ml}>\text{air}$$

$${\text{IR}}_{\text{O}3}=<\text{PFU}/\text{ml}>{\text{O}}_{\text{3}}/<\text{genomes}/\text{ml}>{\text{O}}_{\text{3}}$$

Infectious ratios (IRs) were calculated by dividing mean culture counts (PFU/ml) with mean qPCR values (genomes/ml) for both air and ozone conditions.

$$NIR_{air}=(<PFU/ml>sample-air/<PFU/ml>control-air)\times (<genomes/ml>sample-air/<genomes/ml>control-air)$$

$$NIR_{O3}=(<PFU/ml>sample-O_{3}/<PFU/ml>control-O_{3})\times (<genomes/ml>sample-O_{3}/<genomes/ml>control-O_{3})$$

Each IR was normalized with the nebulizer stocks before each nebulization to ensure that the effects of aerosolization were removed. Normalized infectious ratios (NIRs) were calculated by first dividing the mean sample PFU/ml by the mean control PFU/ml. Then, mean control genomes/ml were divided by mean sample genomes/ml. Finally, both results were multiplied together. NIRs were calculated for ozone and the reference (air) conditions.

$$RIR=NIR\;O_{3}/Med[NIR\;air]$$

Lastly, relative infectious ratios (RIRs) were obtained by dividing each ozone-NIR with the corresponding median air-NIR. This step removed the humidity and aerosol aging effects. As a result, RIRs represent solely the ozone effect for each exposure time and RH.

### Statistical analysis

RIRs were calculated using the traditional formula, except when there was a zero in the numerator of one of the IRs. In these cases, an empirical logit correction was used [71]. This means that 0.5 was added to the numerator, and 1 was added to the denominator. Following the Box-Cox method, logarithm transformations were performed on all RIR values. The following analyses are therefore presented for log(RIR). Two-way ANOVAs were used to test the impacts of humidity and exposure time on RIRs. When significant effects were identified, multiple comparisons were corrected using Tukey’s method. Response surface models (RSMs) were used to identify the best combination of humidity and exposure time, in order to minimize RIRs. First order, second order (for effects with sufficient values) and interaction terms were included in the model. Lack-of-fit tests and R2 statistics were used to identify the relevant effects in order to simplify the models. Contour plots based on the selected models are presented (Figs 3B, 4B and 7B) and enable us to identify the combination of exposure time and humidity that minimizes RIRs.

## Results

### Reference conditions for each virus

**Fig 2:**
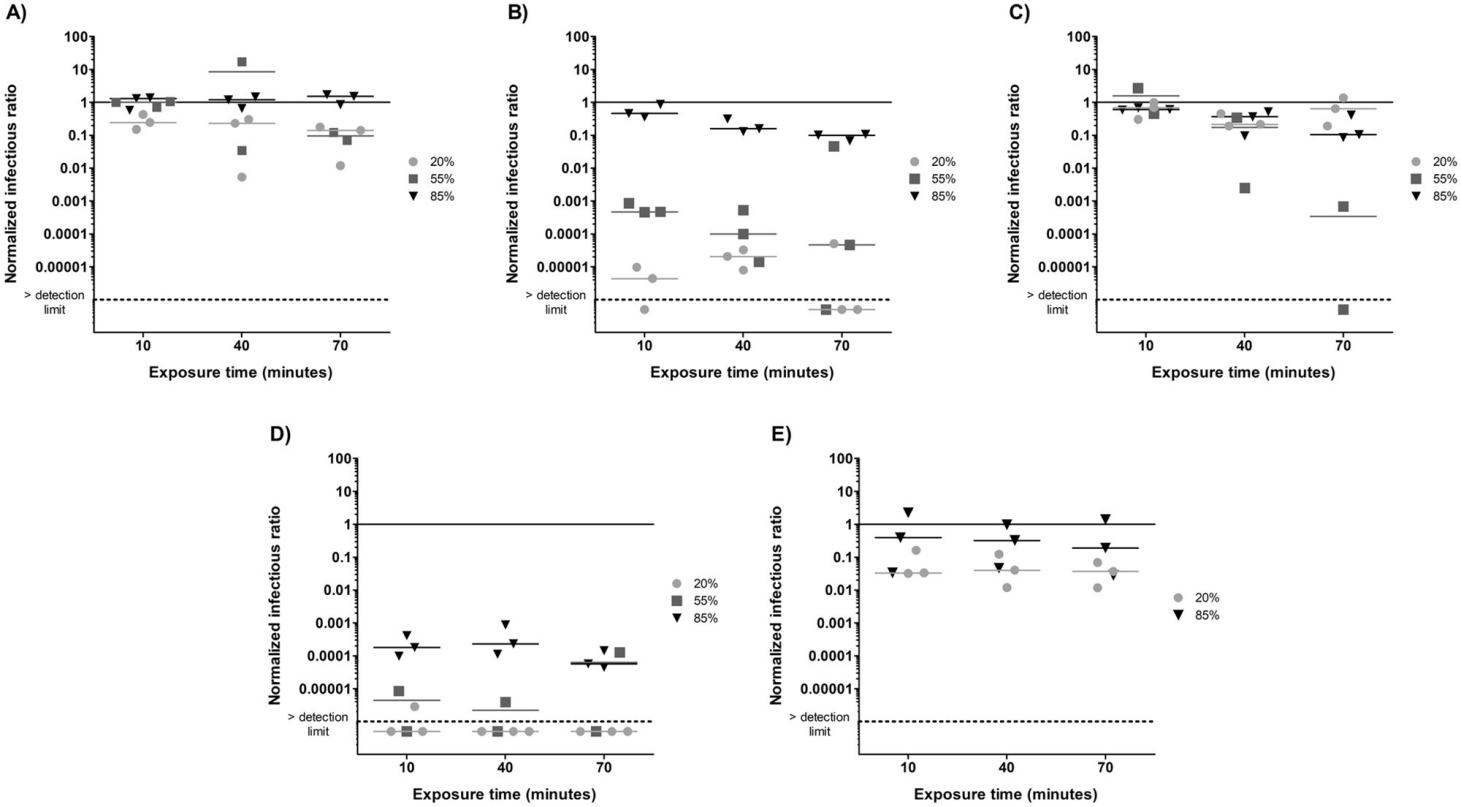
Normalized infectious ratios at three levels of relative humidity and three exposure times for A) φX174, B) PR772, C) MS2 and D) φ6 and two levels of relative humidity and three exposure times for E) MNV-1. The solid line represents the nebulizer content. The dotted line represents the detection limit.

In order to fully appreciate the ozone treatment effects, the reference conditions for each virus are presented in Fig 2. These conditions represent the benchmark effect of exposure to air and humidity as well as the aerosolization and aerosol aging process. An NIR of one means that the infectivity in the samples is the same as that observed in the nebulizer content. An NIR below one indicates that there is a loss of infectivity throughout the reference condition experiment. The horizontal bars represent the median for each RH.

For φX174 (Fig 2A), median NIRs were similar to those of the nebulizer content after a 10 minute and 40 minutes exposure for the three tested RHs. After 70 minutes, there was an order of magnitude decrease at 20% and 55% RH.

For PR772 (Fig 2B), NIRs showed that this phage loses almost all of its infectivity at 20% RH. At 55% RH, there was a decrease of three orders of magnitude after 10 minutes, and four orders after 40 minutes. After 70 minutes, NIR values are dispersed on the graph, but the median NIR is four orders of magnitude below the nebulizer content. At 85% RH, there is only a one order of magnitude decrease after a 70-minute exposure.

For MS2 (Fig 2C), the NIR values were close to one after 10 and 40 minutes for all three RHs. At 70 minutes, there was a decrease of infectivity of one order of magnitude for 85% RH and three orders of magnitude for 55% RH.

The φ6 virus (Fig 2D) was no longer infectious at 20% RH when exposed to the reference conditions. Therefore, the additional effect of ozone could not be assessed at this RH. The NIRs at 55% RH are also very low, with one replicate below the detection limit for each exposure time. At 85% RH, there was a decrease of infectivity of more than three orders of magnitude after 10 and 40 minutes and four orders of magnitude at 70 minutes.

MNV-1 (Fig 2E) infectivity decreased by one order of magnitude at 20% RH but was resistant at 85% RH with NIR values similar to those of the nebulizer content.

### Ozone effect in air sampler

Mean RIRs and standard deviation for each phage from the ozone in the air sampler control experiments were the following: 5.12 ± 8.24 for φ6, 0.55 ± 0.71 for φX174, 0.77 ± 0.54 for PR772 and 4.48 ± 6.47 for MS2. Ozone has a virucidal effect if the RIR is below one. Results from the current study show that ozone has no effect in the sampling liquid, therefore there is no need to apply a mathematical correction for ozone effect in the air sampler.

### Relative infectious ratios

**Fig 3:**
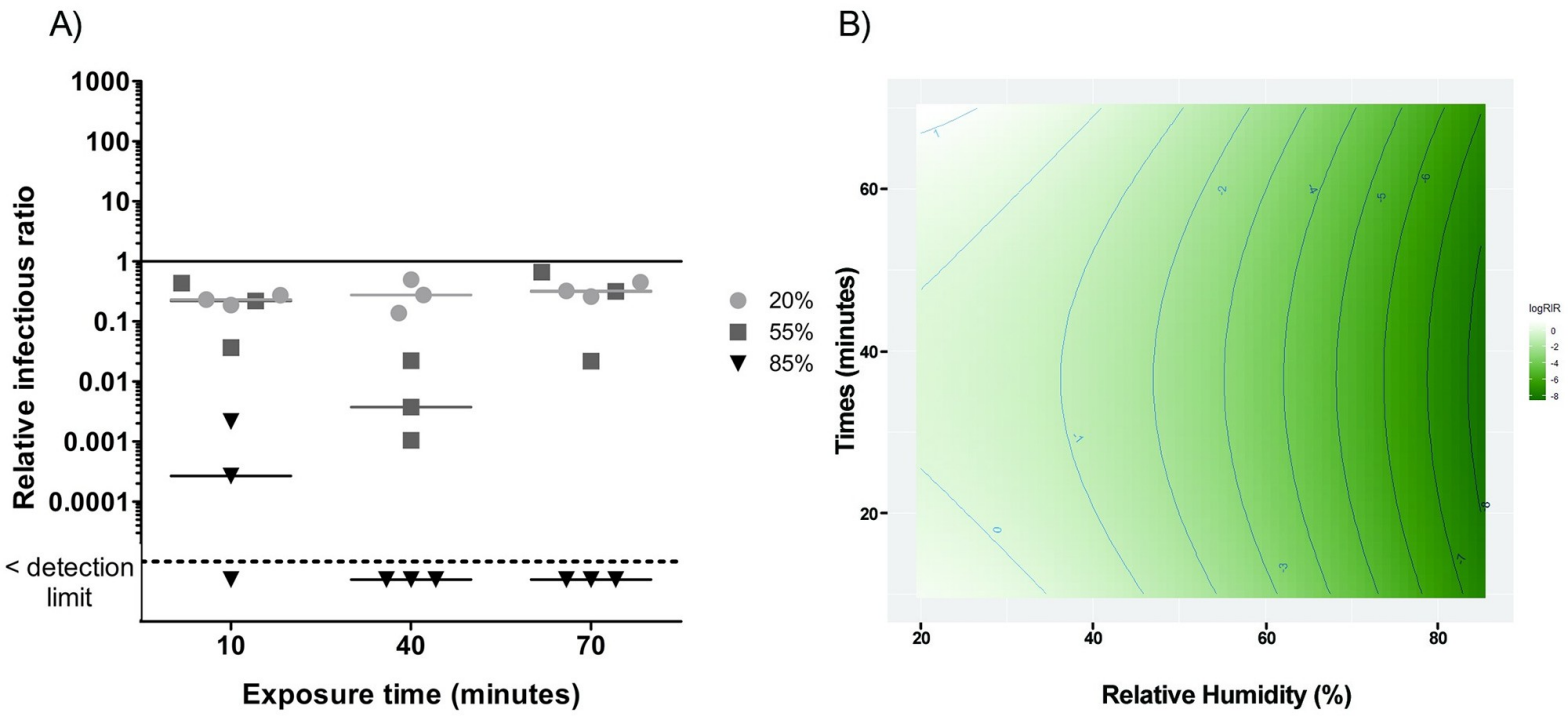
A) Ozone effect on phage φX174 infectivity at three levels of relative humidity and three exposure times. The solid line represents the reference value without ozone. The dotted line represents the detection limit. 20% RH values are represented by circles (●), 55% RH by squares (■) and 85% RH by triangles (▼). B) RSM between exposure time and humidity percentages for φX174. The darker the green colour, the greater the inactivation related to relative humidity and time combination.

**Fig 4:**
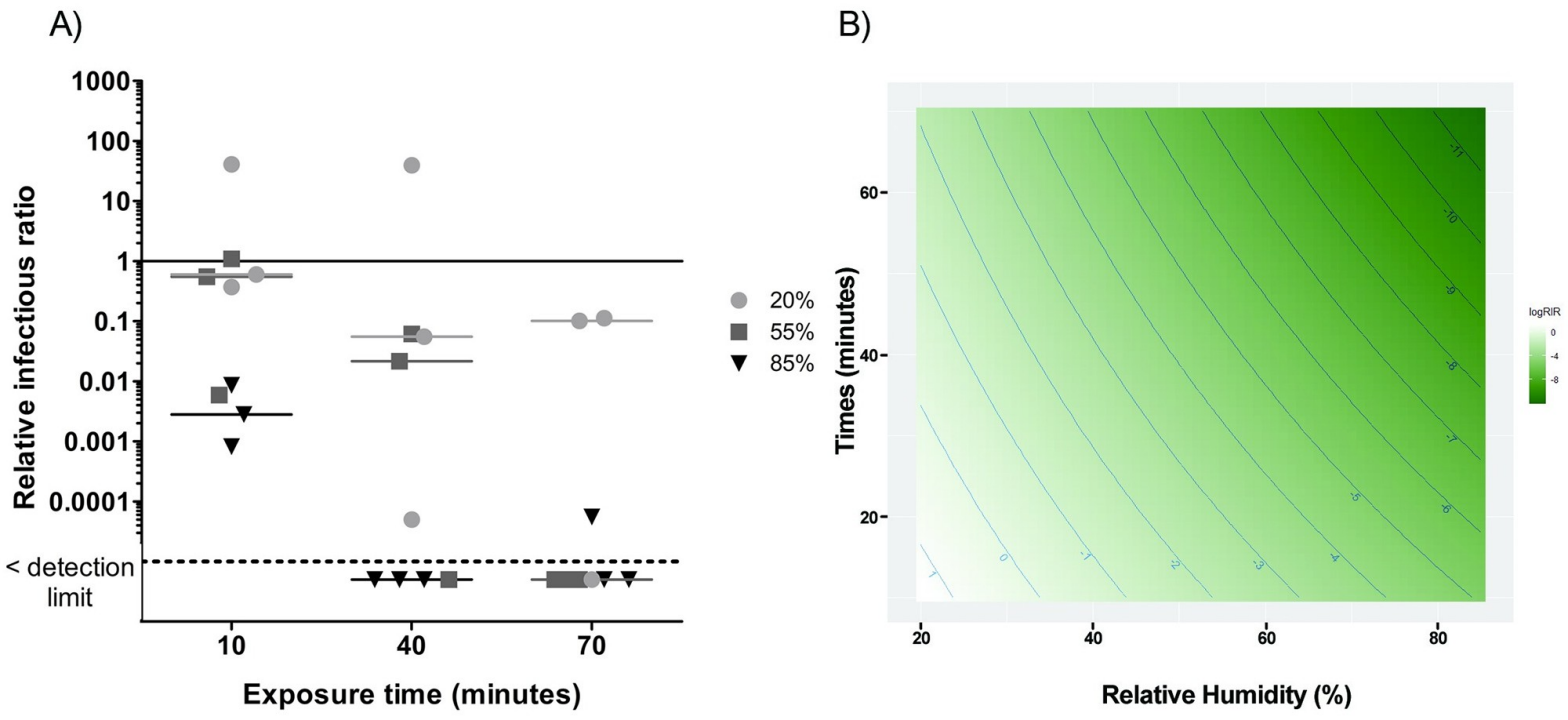
A) Ozone effect on phage PR772 infectivity at three levels of relative humidity and three exposure times. The solid line represents the reference value without ozone. The dotted line represents the detection limit. 20% RH values are represented by circles (●), 55% RH by squares (■) and 85% RH by triangles (▼). B) RSM between exposure time and humidity percentages for PR772. The darker the green colour, the greater the inactivation related to relative humidity and time combination.

**Fig 5:**
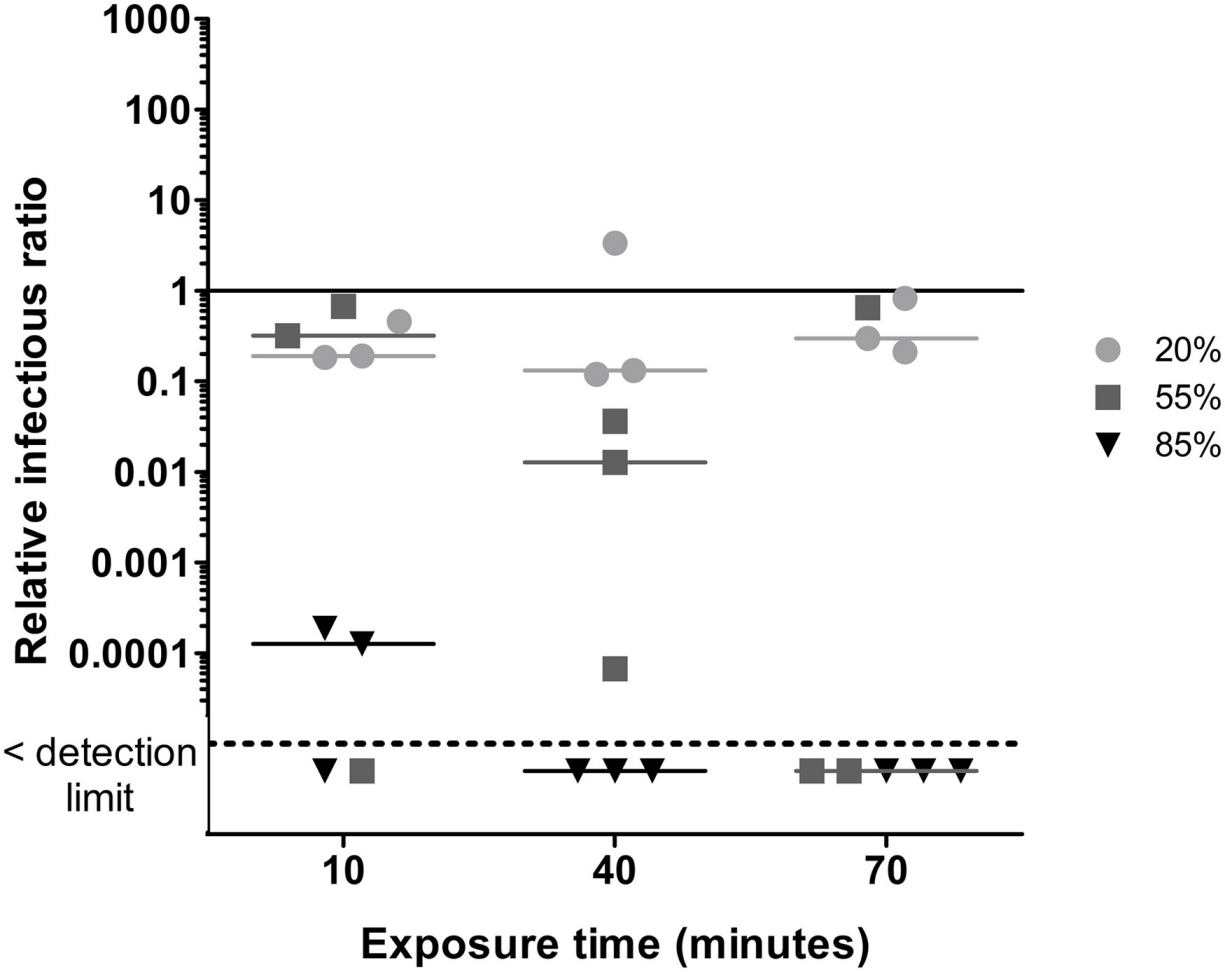
Ozone effect on phage MS2 infectivity at three levels of relative humidity and three exposure times. The solid line represents the reference value without ozone. The dotted line represents the detection limit. 20% RH values are represented by circles (●), 55% RH by squares (■) and 85% RH by triangles (▼).

**Fig 6:**
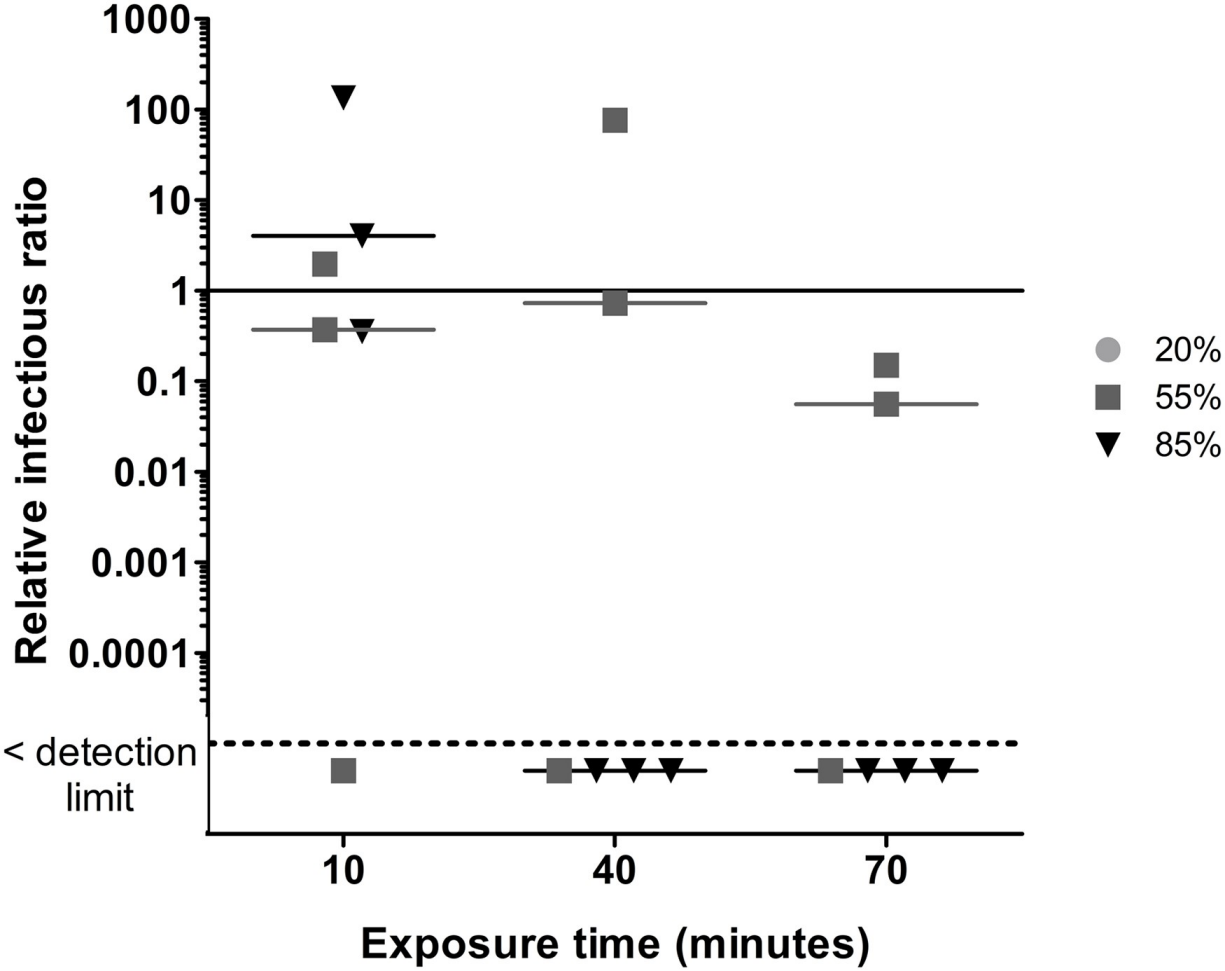
Ozone effect on phage φ6 infectivity at three levels of relative humidity and three exposure times. The solid line represents the reference value without ozone. The dotted line represents the detection limit. 20% RH values are represented by circles (●), 55% RH by squares (■) and 85% RH by triangles (▼).

**Fig 7:**
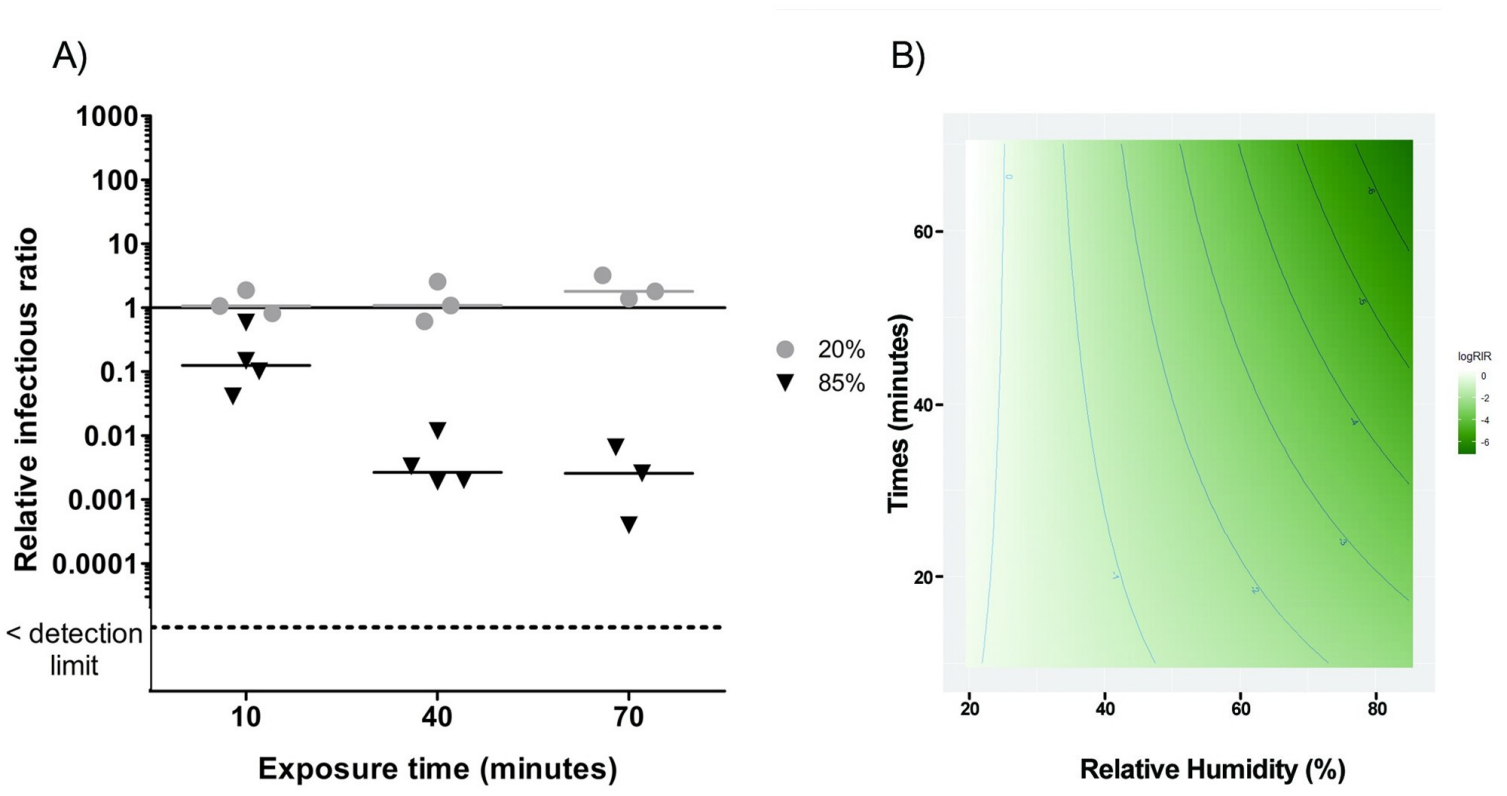
A) Ozone effect on MNV-1 infectivity at two levels of relative humidity and three exposure times. The solid line represents the reference value without ozone. The dotted line represents the detection limit. 20% RH values are represented by circles (●) and 85% RH by triangles (▼). B) RSM between exposure time and humidity percentages for MNV-1. The darker the green colour, the greater the inactivation related to relative humidity and time combination.

Figs 3–7 present RIRs for phages and MNV-1 obtained with ozone treatment at 1.13 ppm ± 0.26 ppm and 0.23 ppm ± 0.03 ppm, respectively. RIRs represent the effect of ozone only, since data were corrected for the effect of RH and aerosol aging without ozone. Therefore, the overall treatment effects are due to the addition of the reference conditions (Fig 2) to the exposure to ozone (Figs 3–7).

#### φX174

The RIRs for φX174 (Fig 3A) are close to the reference value at 20% RH. At 55% RH, RIRs decreased by between one and two orders of magnitude. The effects of ozone are much greater at 85% RH. Indeed, there is an immediate virucidal effect (decreases of 3 to 4 orders of magnitude) and the ratios fell below the detection limit after 40 minutes of exposure. The interaction between RH and exposure time is significant (p = 0.02) for φX174. The RSM analysis (Fig 3B) reveals that the best virucidal effect is obtained with a high RH (above 80%) no matter the exposure time. Therefore, the inactivation of φX174 can be achieved using a combination of 1.13 ppm ± 0.26 ppm of ozone and high RH, but the exposure time can be as short as 10 to 40 minutes.

#### PR772

With PR772 (Fig 4), it was not possible to conclude if ozone has a virucidal effect at 20% RH, but there was a gradual decrease in infectivity at 55% RH with RIRs below the detection limit after 70 minutes of exposure. At 85% RH, there was an immediate decrease of two to three orders of magnitude and RIRs dropped under the detection limit after an exposure of 40 minutes. At 70 minutes, two out of three replicates were below the detection limit. A significant interaction between RH and time was also observed for PR772 (p < 0.01). Adjusted R^2^ was 0.77. The efficacy of PR772 inactivation was better visualized with the RSM graph, which shows that a concentration of 1.13 ppm ± 0.26 ppm of ozone has a stronger virucidal effect at high RH and a long exposure time.

#### MS2

For MS2 (Fig 5), RIRs at 20% RH are close to the reference value. There was a gradual increase of the virucidal effect when ozone was used at 55% RH. At 85% RH, there was a strong decrease after 10 minutes and RIRs fell below the detection limit after 40 minutes. The quantification of total viruses (qPCR) for MS2 was problematic for some samples, which resulted in missing values. Therefore, the interaction between time and RH were assessed separately. At 20% RH, the interaction between time and relative infectious status was not significant (p = 0.16). There was a small but insignificant interaction at 55% RH (p = 0.08) and a significant interaction at 85% RH (p < 0.01).

#### φ6

The 20% RH values are not shown in Fig 6 because the IRs for the reference conditions were already below the detection limit (Fig 2D). Indeed, no plaque counts were observed for these conditions, which resulted in IRs equal to zero. Therefore, it was impossible to calculate NIRs and RIRs. These conditions were also excluded from statistical analyses and RSM analysis. The ozone effect at 55% RH was not significant, but was significant at 85% RH since RIRs were below the detection limit after a 40-minute exposure. Statistical analysis shows that the interaction of RH and time is significant (p < 0.01). The adjusted R^2^ was 0.94.

#### MNV-1

For MNV-1 (Fig 7), ozone had no effect at 20%. At 85% RH, there was an immediate decrease of one order of magnitude and then a decrease of two additional orders of magnitude at 40 minutes. The length of time and RH interaction is highly significant (p < 0.01). The adjusted R^2^ was 0.87. The RSM graph shows that the ozone effect at a concentration of 0.23 ppm ± 0.03 ppm is maximized at high RH with a longer exposure time.

### Overview of ozone efficacy for aerosolized viruses

The ozone exposures that yielded an inactivation of at least two orders of magnitude for each virus at three levels of relative humidity and three exposure times are summarized in Table 3. At 20% RH, no additional treatment resulted in an inactivation of two orders of magnitude. PR772 and φ6 were already close or below the detection limit when exposed to the reference condition (Fig 2B and 2D), therefore no additional effect could be recorded when exposed to ozone. At 55% RH, a 40-minute exposure was required for φX174 and MS2 inactivation. At 85% RH, 10 minutes were required for φX174, PR772 and MS2. The φ6 and MNV-1 viruses showed inactivation levels of at least two orders of magnitude after 40 minutes.

**Table 3:** Summary of the effect of ozone at 1.13 ppm ± 0.26 ppm on the four tested phages and at 0.23 ppm ± 0.03 ppm on MNV-1 at three levels of relative humidity and three exposure times.

Exposure time (minutes)	Relative humidity (%)		
	20	55	85
10	PR772* φ6*	-	φX174 PR772 MS2
40	-	φX174 MS2	φ6 MNV-1
70	-	-	-

The inactivation of at least two orders of magnitude are shown. No growth when exposed to the reference condition (Fig 2B and 2D).

## Discussion

This study assessed the inactivation of airborne viruses using 1.13 ppm ± 0.26 ppm and 0.23 ppm ± 0.03 ppm of ozone at various levels of RH and exposure times. To date, only a few studies have used low ozone concentrations for this purpose, therefore there is a need to evaluate the effects of this kind of air treatment given its potential for hospitals using natural ventilation.

Using lower ozone concentrations is less costly because a high capacity ozone generator is not required. Ozone concentrations of below 0.1 ppm may be feasible to treat the air inside unoccupied hospital rooms. According to the Quebec Occupational Health and Safety Organization (Commission des normes, de l’équité, de la santé et de la sécurité du travail [CNESST]) respiratory protective equipment is not needed when the ozone concentration inside a room does not exceed the threshold value of 0.1 ppm. However, because this gas is harmful to humans at concentrations above this value, patients and staff should not be present during air treatment in case the concentration exceeds 0.1 ppm. When using lower ozone concentrations, longer exposure times are required. To test this the environmental rotating drum was used because it allowed for longer viral aerosol exposure times.

Another element to consider before implementing an air treatment plan involving ozone inside naturally ventilated rooms is the evaluation of the pressure inside the rooms. Negative pressure would prevent ozone leakage through the doors, but the majority of hospital rooms do not have this feature. Therefore, testing must be conducted for possible ozone leakage when doors are closed in order to evaluate the treatment’s feasibility. For better protection, ozone destructors can be used and operated in the hallway near the closed door of the hospital rooms and inside them when the treatment is completed. Treating the air directly in the heating, ventilation and air-conditioning (HVAC) plenum with the help of ozone destructors is also of interest. The recycled air would be clean and ozone-free, allowing people to stay inside the treated rooms during continuous air treatment. Higher ozone concentrations could also be used in HVAC plenum, resulting in faster inactivation of airborne viruses. The main drawback of this installation is that, contrary to the *in situ* air disinfection protocol, surfaces would not benefit from additional decontamination. Based on the constraints associated with the type of ventilation, a decision must be made about whether one or a combination of both methods best fits the available infrastructure. Low capacity ozone generators and ozone destructors are quiet, inexpensive and easy to use; these devices should be easily supported for use in hospitals and other public environments.

Studies have demonstrated that the presence of ozone under high RH conditions leads to the formation of more radicals than in dry air [52, 64, 72]. Tseng and Li (2006) [52] observed that the inactivation of phages is increased when high RH (85%) is used, which is consistent with our findings. As seen in Table 3, with the exception of PR772 and φ6, an exposure time of at least 40 minutes at 85% RH is most effective for the inactivation of the other viruses using ozone.

Tseng and Li (2006) have also used low ozone concentrations, but for short time periods, resulting in lower inactivation rates that the present work [52]. Indeed, for MS2 and φ6 we obtained RIRs below the detection limit compared to a reduction of 1 and 2 orders of magnitude, respectively. For φX174, there was a decrease of 3 to 4 orders of magnitude instead of 1 to 2. Therefore, a lower inactivation rate when using low ozone concentrations can be overcome when increasing the air treatment time.

The results in Fig 2 show that viruses have different tolerances for various RH levels. PR772 and φ6 lose almost all of their infectivity under the reference conditions at 20% RH. The same applies for φ6 at 55% RH. Therefore, the most effective inactivation conditions for those species do not require ozone. The results presented in Fig 2 are comparable with those of Turgeon *et al*. (2016) [45] and Verreault *et al*. (2015) [58], who used the same environmental chamber setup. The only notable difference is with φ6 at low RH, which did not become inactivated even after a 2-hour exposure to the reference conditions [45]. It remained infectious after a 6-hour and a 14-hour exposure, but there was more variability in RIR replicates [58]. Because there seems to be a great variation of φ6 infectivity due to the experimental conditions, calculating RIRs allows for the removal of the aerosolization and RH effects of these experimental conditions.

As suggested in a study assessing the infectivity conservation of airborne Influenza at various humidity levels, fluid composition could affect the survival of viruses contained in aerosols [73]. A simplified human body fluid contains salts, proteins and lipids [74], which could all interact with ozone [75]. In further studies, it would be of interest to measure the survival of airborne viruses in presence of human fluid when exposed to ozone. These results could then be compared to the experiments using phage buffer presented in this study and give additional insight regarding the efficacy of ozone treatment.

Additional investigations would also be needed regarding the interaction of ozone with other compounds found in hospital rooms, some of them released from furnishings and cleaning products. As shown by Nazaroff and Weschler (2004), ozone reacts with terpenes found in these type of products, leading to the formation of secondary pollutants [76]. Assessing the potential exposure risks to these pollutants would be of great interest.

Even if some viruses may not survive in dry air, air humidification prior to ozone treatment in hospital rooms should be considered for the inactivation of the remaining infectious viruses. This humidification could reduce treatment time and result in a better overall efficacy. In addition, other environments could benefit of an air treatment to prevent subsequent viral outbreaks, for example classrooms or cruise ships after a norovirus outbreak.

## Conclusion

The results obtained in this study demonstrate the efficacy of an air treatment for phage and MNV-1 inactivation using low ozone concentrations, 1.13 ppm ± 0.26 ppm and 0.23 ppm ± 0.03 ppm, respectively, at various RH levels and exposure times of up to 70 minutes. An exposure of 40 minutes at 85% RH yields the inactivation of at least two orders of magnitude for φX174, MS2 and MNV-1. An exposure to the reference conditions at 20% RH for 10 minutes for PR772 and φ6 was enough to yield the same results. The inactivation of other problematic viruses should be tested to obtain supplementary evidence regarding this air treatment and with the eventual possibility of implementing it in hospital settings. Since Influenza is an enveloped virus, it would be interesting to evaluate if the treatment efficacy is the same as its surrogate phage, φ6. In the context of the SARS-CoV-2 pandemic, future work is needed to assess the efficacy of an ozone treatment in order to reduce the transmission of this virus in hospital settings and other indoor public spaces. This treatment could also be tested with bacteria resistant to antibiotics, including *Clostridium difficile*, methicillin-resistant *Staphylococcus aureus* and vancomycin-resistant enterococci, which are serious threats to hospitalized patients. Lastly, low ozone concentrations could be used for air treatment inside hospital rooms ventilated naturally, providing an additional tool for hospitals that do not possess HVAC plenums.
