# Correction: Ozone efficacy for the control of airborne viruses: Bacteriophage and norovirus models

**DOI:** 10.1371/journal.pone.0334631

**Published:** 2025-10-14

**Authors:** Marie-Eve Dubuis, Nathan Dumont-Leblond, Camille Laliberté, Marc Veillette, Nathalie Turgeon, Julie Jean, Caroline Duchaine

The following information is missing from the Funding statement: CD: Institut de recherche Robert-Sauvé en santé et sécurité du travail (IRSST 2017-0004).
